# Investigation of the Performance of HEMT-Based NO, NO_2_ and NH_3_ Exhaust Gas Sensors for Automotive Antipollution Systems

**DOI:** 10.3390/s16030273

**Published:** 2016-02-23

**Authors:** Yacine Halfaya, Chris Bishop, Ali Soltani, Suresh Sundaram, Vincent Aubry, Paul L. Voss, Jean-Paul Salvestrini, Abdallah Ougazzaden

**Affiliations:** 1 Georgia Tech Lorraine, Georgia Tech- Centre National de la Recherche Scientifique (CNRS), Unité Mixte Internationale (UMI 2958), 2-3 rue Marconi, Metz 57070, France; yacine.hal@gmail.com (Y.H.); cbishop@georgiatech-metz.fr (C.B.); suresh9111@gmail.com (S.S.); paul.voss@ece.gatech.edu (P.L.V.); salvestr@metz.supelec.fr (J.-P.S.); 2 Peugeot Citroën PSA, 75 Avenue de la Grande Armée, Paris 75116, France; vincent.aubry@mpsa.com; 3 School of Electrical and Computer Engineering, Georgia Institute of Technology, Atlanta, GA 30332, USA; 4 Institute d’Electronique, de Microélectronique et de Nanotechnologie, Centre National de Recherche Scientifique (IEMN/CNRS) 8520, Université de Lille Science et technologies, Villeneuve d’Ascq 59652, France; ali.soltani@iemn.univ-lille1.fr; 5 Université de Lorraine, LMOPS EA 4423, 2 rue E. Belin, 57070 Metz, France

**Keywords:** AlGaN/GaN heterostructure, HEMT transistor, NO_*x*_ and NH_3_, automotive exhaust line, gas sensor

## Abstract

We report improved sensitivity to NO, NO${}_{2}$ and NH${}_{3}$ gas with specially-designed AlGaN/GaN high electron mobility transistors (HEMT) that are suitable for operation in the harsh environment of diesel exhaust systems. The gate of the HEMT device is functionalized using a Pt catalyst for gas detection. We found that the performance of the sensors is enhanced at a temperature of 600 °C, and the measured sensitivity to 900 ppm-NO, 900 ppm-NO${}_{2}$ and 15 ppm-NH${}_{3}$ is 24%, 38.5% and 33%, respectively, at 600 °C. We also report dynamic response times as fast as 1 s for these three gases. Together, these results indicate that HEMT sensors could be used in a harsh environment with the ability to control an anti-pollution system in real time.

## 1. Introduction

The need to meet upcoming EURO7 emission standards for diesel exhaust systems provides the motivation for improved sensor technologies. Many anti-pollution systems that limit total NO${}_{x}$ (NO + NO${}_{2}$) emissions require the injection of NH${}_{3}$, in the form of urea, into the selective catalytic reduction system (SCR) in order to reduce NO and NO${}_{2}$ emissions into N${}_{2}$ and H${}_{2}$O [1]. The amount of ammonia necessary for optimal elimination of NO and NO${}_{2}$ essentially depends on the ratio between the concentration of NO${}_{2}$ and NO present in the exhaust gas. However, currently-used NO${}_{x}$ gas sensors cannot give accurate information on the NO${}_{2}$/NO ratio. It is instead calculated using a predictive model. The current response time to achieve 90% of stationary response to NO${}_{2}$ (for 10–1650 ppm) is around 2 s, and the sensors cannot detect NH${}_{3}$ [2,3,4]. Therefore, there is a need for improved measurement of NO, NO${}_{2}$ and NH${}_{3}$ gas pollutants by: (1) increasing the sensitivity of NO${}_{x}$ sensors, with the ability to operate in a concentration range of 10–2000 ppm; (2) detecting ammonia in order to avoid overconsumption of urea and subsequent ammonia pollution; and (3) decreasing response time to less than 2 s. These improvements will allow more precise control of the anti-pollution system and better fuel efficiency while meeting the new regulations.

Among various approaches studied to meet these requirements, sensors based on semiconductor materials have significant advantages compared to electrochemical-based sensors. They have shown the ability to detect several types of pollutants (both oxidizing and reducing gases) [5,6,7,8], have low cost and can be assembled in arrays. The Group III nitrides exhibit high resistance to corrosion and high thermal and chemical stability [9] as compared to silicon, which make them potential solutions for automotive exhaust gas sensors. It has been shown that GaN Schottky diodes with catalytic active materials, such as platinum (Pt) and palladium (Pd), as electrodes [10,11,12,13] can be used to detect NO, NO${}_{2}$ and NH${}_{3}$ using different structures: metal-semiconductor (MS) [14,15], metal-oxide-semiconductor (MOS) [7] and metal-semiconductor-metal (MSM) [16]. Such sensors have been shown to be sensitive to a small range of (NO, NO${}_{2}$) concentrations of 10–100 ppm [7], to have fast response time of 5 s using the BGaN-SL structure for NO${}_{2}$ detection [16] and the possibility of operation at 400 °C [14,15]. However, these studies do not show the possibility of the detection of the three target gases using a single device.

To improve the performance of NO${}_{x}$ gas sensors, we choose to use AlGaN/GaN high electron mobility transistors (HEMTs) because of a number of advantages, as compared to Schottky diodes. These include a larger current than that of Schottky diodes, which yields lower theoretical detection limits. In addition, HEMTs allow for the possibility of adjusting the gate bias to optimize sensitivity, which makes them more robust for automotive applications. However, to date, few studies have been reported for NO, NO${}_{2}$ and NH${}_{3}$ using this type of sensor. Sensitivity to NH${}_{3}$ [6] has been reported with a detection limit of 35 ppm at room temperature. Another study done at an operation temperature of 400 ${}^{\circ }$C using the same type of sensors [17] reported sensitivity to NO${}_{2}$; however, no sensitivity to NO was reported. The sensors in these studies show a limit in their performance in terms of sensitivity and the possibility to detect all three gases NO, NO${}_{2}$ and NH${}_{3}$. Furthermore, they do not show any information about the behavior of these sensors at an operating temperature higher than 400 °C, which is often the case in a diesel exhaust line. To overcome these issues, Pt-AlGaN/GaN sensors have been modeled, simulated and experimentally tested for different operation temperatures, from 300 °C–600 °C, to the three target gases. In this paper, we focus on three main topics: First, we show that using an optimized design allows one sensor to detect all three target gases with a large increase in sensitivity. Second, we present experimental results in conditions similar to the real case test in the exhaust line, with measurements of the ability of the sensors to follow the change in gas concentrations for short time pulses from 10 downto 1 s in length. Finally, we present the characterization of our sensors at high operation temperature (up to 600 °C), followed by a comparison of the sensitivity and response time for both cases (300 °C and 600 °C).

## 2. Experimental Section

### 2.1. Operating Principles of AlGaN/GaN HEMT-Based Gas Sensors

The AlGaN/GaN heterojunction is characterized by the formation of a two-dimensional electron gas (2DEG) at the AlGaN/GaN interface. The variation of the concentration of the free carrier charges, which represent the change in the drain-source current when the drain-source potential is held constant, is strongly linked to the variation of the potential beneath the gate (at the interface between the gate and the AlGaN layer). The drain-source current is given as a function of applied gate voltage in Equation (1), which is derived from the self-consistent solution of the 1D Poisson and Schrodinger Equations [18]:

(1) $$I_{ds}=\frac{\varepsilon W_{g}}{d}\left[\nu \left(x\right)\left(V_{gs}-V_{th}-E_{F}\right)\right]$$

where *ε* is the permittivity of the Al${}_{m}$Ga${}_{1-m}$N layer, $W_{g}$ is the gate width, *d* is the thickness of the AlGaN layer, ν(x) is the carrier velocity at position x in the channel, *V${}_{gs}$* is the applied gate-to-source bias, *V${}_{th}$* is the threshold voltage when the device turns off and $E_{F}$ is the Fermi level with respect to the GaN layer conduction band.

According to this equation, the gate voltage has a direct effect on the channel current $I_{ds}$. The contact between the gate and the AlGaN layer is a Schottky contact that is characterized by a difference in electrostatic potential due to the difference in the work functions of these two materials, which leads to the formation of a space charge region beneath the gate. Therefore, any variation in the gate bias $V_{gs}$ induces a change in the depth of the potential well at the AlGaN/GaN heterojunction through the space charge region and, thus, a change in the measured current $I_{ds}$.

**Figure 1:**
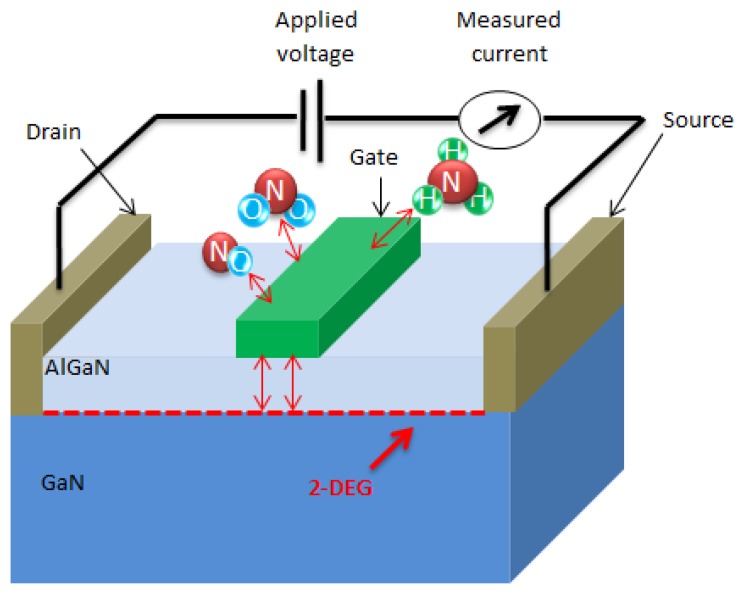
Detection mechanism of the AlGaN/GaN high electron mobility transistors (HEMT)-based gas sensor.

In the case of an HEMT used as a gas sensor, one takes advantage of the fact that interface states exist at the interface between the gate metal (Pt) and semiconductor (AlGaN layer), which has a direct effect on the space charge region beneath the gate. The adsorption of the gaseous molecules on the functional layer, as shown in Figure 1, leads to changes in the surface depletion layer [19] that affect the number of mobile carriers in the 2DEG of the HEMT structure, resulting in a large change of the output current of the device. The three target gases used in this study show different detection mechanisms. The NO${}_{2}$ molecules dissociate on the Pt layer into oxygen ions that diffuse through grain boundaries to the AlGaN surface. NO, on the other hand, is more chemically stable and does not readily dissociate into oxygen ions; however, the NO gas molecules must interact directly with traps either at the AlGaN surface or capacitively through pores in the Pt layer. NH${}_{3}$ can also be dissociated by the Pt catalytic metal leading to hydrogen ions that diffuse to the Pt-AlGaN interface forming a hydrogen dipolar layer. It has also been suggested that NH${}_{3}$ can also follow a second detection mechanism, where it is capacitively linked to the interface traps through pores in the functional layer [6,15,20].

In this way, the HEMT is sensitive to the gas molecules that induce a measurable current change in the HEMT. In our case, the drain-source bias ($V_{ds}$) is fixed at 5 V without any bias applied on the gate ($V_{gs}$ = 0 V). In the following sections, we will explain the characterization of the sensors using operating temperatures of 300 °C and 600 °C.

### 2.2. Description of the Gas Sensor Fabricated and the Test Method Used

The AlGaN/GaN heterostructure was grown by metal organic vapor phase epitaxy (MOVPE) on a semi-insulating GaN template on sapphire using trimethylgallium (TMGa), trimethylaluminum (TMAl) and ammonia (NH${}_{3}$) as the gallium, aluminum and nitrogen sources, respectively.

**Figure 2:**
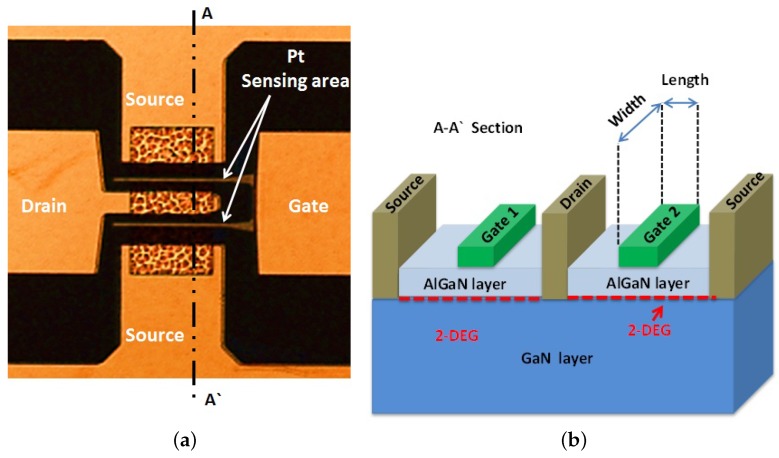
(**a**) Processed HEMT sensor device; (**b**) cross-section of the AlGaN/GaN HEMT structure.

The epilayers consist of a 3.5-μm Fe-doped semi-insulating GaN on sapphire substrate, a 260-nm undoped GaN layer and a 19-nm undoped AlGaN layer with an aluminum (Al) composition of 30%, which was verified by XRD measurements. AFM measurements show a smooth surface with RMS roughness of 0.77 nm and a V-defect density of around 1 × 10${}^{8}$ cm${}^{2}$. After lithography, Pt was evaporated with a thickness of 15 nm followed by liftoff to obtain more than 60 sensors in one chip of a 1-cm${}^{2}$ size. The Pt gate, which is the sensing area, has different dimensions (length and width). The HEMT structure and processed device are shown in Figure 2.

**Figure 3:**
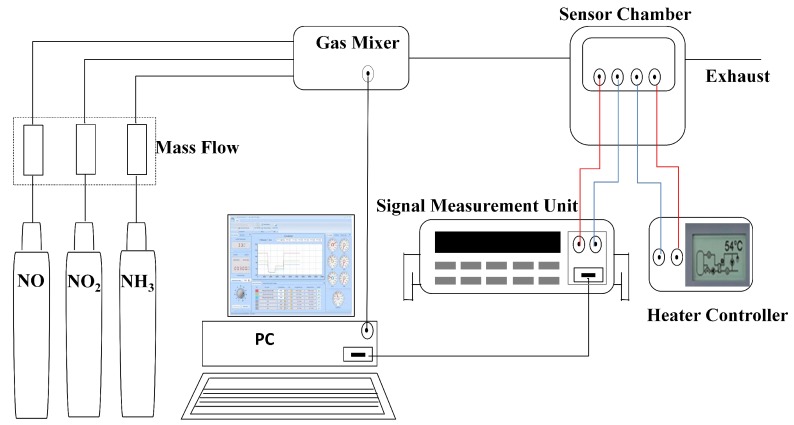
Experimental setup of the HEMT gas sensor for NO${}_{x}$ and NH${}_{3}$ detection.

For NO, NO${}_{2}$ and NH${}_{3}$ sensing tests, the sensors were loaded in a closed chamber containing a ceramic hot plate to control the operating temperature and were connected to a Keithley 236 IV measurement system, as shown in Figure 3.

Gas sources consist of pure N${}_{2}$ as the reference gas and NO, NO${}_{2}$ and NH${}_{3}$ gas sources diluted into N${}_{2}$. Pressure regulators were connected to the testing chamber via a gas blender, so that the pressure, concentration and flow rate were controlled during the measurements. A flow rate of 100 sccm was used, and with all external factors controlled, we can attribute any changes in the steady-state signal to the gas detection mechanism previously described. For each measurement, the signal under pure N${}_{2}$ was used as a reference for comparison with the signal under the test gas diluted into nitrogen. The sensing characteristics were tested at an operating temperature that can range from room temperature to 600 °C using an external temperature controller. We have demonstrated previously that these sensors can reset to their initial current under pure nitrogen N${}_{2}$ at an operating temperature up to 280 °C for each of the three target gases. In the present investigation, the operating temperatures used are 300 °C and 600 °C.

## 3. Results and Discussion

### 3.1. Performance Enhancement of a Pt-AlGaN/GaN HEMT-Based Sensor Using an Optimized Design

**Figure 4:**
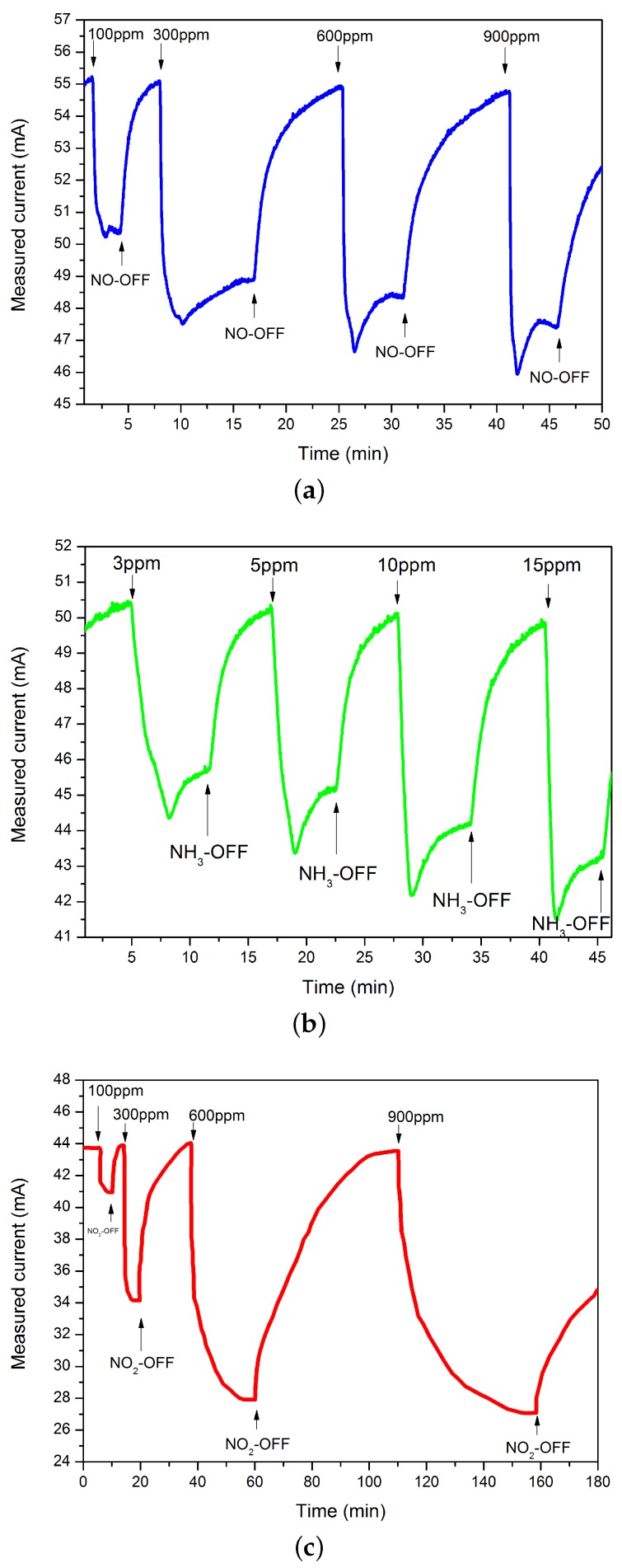
Response of the Pt-AlGaN/GaN HEMT sensor to (**a**) NO; (**b**) NH${}_{3}$; and (**c**) NO${}_{2}$ gases for different concentrations using sensors with a gate size of 2 μm × 150 μm.

Several sensors were fabricated on the same sample (containing more than 60 sensors) with different designs. The following results show an example of the behavior for one sensor that has a gate size of L × W = 2 m × 150 m. Figure 4 shows the transient response and recovery curves of Pt-AlGaN/GaN HEMT sensors to the three target gases, NO, NO${}_{2}$ and NH${}_{3}$, for different concentrations in pure nitrogen as carrier gas at 300 °C. Exposure of the sensor to the gases leads to decreased source-drain current with a significant change even for low concentrations of NO and NO${}_{2}$ (100 ppm) and at concentrations as low as 3 ppm for NH${}_{3}$. From these curves, we calculate the sensitivity, a parameter to quantify the performance of the sensors, using:

(2) $$S=\frac{I_{N2}-I_{gas}}{I_{gas}}$$

where $I_{N2}$ and $I_{gas}$ are the drain current levels at steady state under flows of N${}_{2}$ and the test gas, respectively.

For NO detection, the drain current initially decreased from 54.5 mA down to 46 mA, followed by an increase until stability at 47.5 mA after a given time. An absolute current change of 7 mA was measured by switching the gas source from N${}_{2}$ to 900 ppm of NO/N${}_{2}$. The sensor shows the same trend as NO detection of decreasing and increasing the drain current when it was exposed to 15 ppm NH${}_{3}$/N${}_{2}$. It showed a large absolute current change of 4 mA. The sensitivity for these two gases corresponds to 12.8% and 13%, respectively. The trend for NO${}_{2}$ detection is opposite the ones explained before. The current continues to decrease when the sensor is exposed to NO${}_{2}$ until reaching steady state. For 900 ppm NO${}_{2}$/N${}_{2}$, the absolute change in output current $I_{ds}$ was 14 mA. The sensitivity corresponds to 33%. We observe some saturation of sensitivity for high concentrations of NO${}_{2}$, which may be due to nearly complete interface coverage. Opposite trends were typically expected for oxidizing and reducing gases. According to the curves shown in Figure 4, we observe that the sensor behaves differently in the presence of each gas. The current under NO${}_{2}$ gas decreases until steady state, corresponding to a negative surface potential at the Pt/AlGaN interface.

However, for NO and NH${}_{3}$ gases, the trend observed may be attributed to a second reaction mechanism with a different response time or it may again be related to the number of available interface traps, since the behavior depends on the concentration of gas. Furthermore, and according to the literature, there may be other detection mechanisms for NO [14,21] and NH${}_{3}$ [22,23,24] depending on the temperature and other effects. For example, NO is more chemically stable and does not readily dissociate into oxygen ions; however, it must interact directly with traps either at the AlGaN surface or capacitively through pores in the Pt layer, which is a different detection mechanism compared to NO${}_{2}$.

According to these results, the sensors give a wide dynamic range for both NO and NO${}_{2}$ with high sensitivity and show the possibility to detect NH${}_{3}$ for very low concentrations (3 ppm–15 ppm), compared to other reports [6,17]. The results also show that the sensitivity increases with the concentration of the gas. This enhancement of sensitivity of our sensors compared to similar devices reported in the literature may be due to the gate size, which has been optimized using extensive analytical modeling techniques. Furthermore, this enhancement may be due to the thickness of the functional layer used in our design (15 nm), which facilitates the chemical reaction of the gas molecules, leading to an increase in the sensitivity and a decrease in the response time. Performance also depends on the operating temperature, which will be discussed in detail in the following section. The transient measurements presented for the three gases represent the change in the response and recovery of the sensor from the initial value (under nitrogen) to the final value (steady state) under the effect of the test gas.

**Figure 5:**
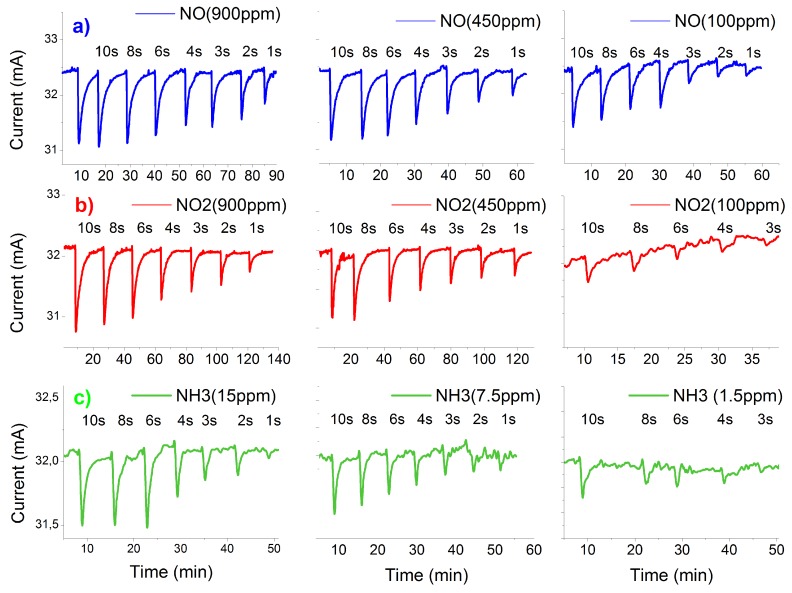
Sensors’ response under different exposure times for (**a**) NO; (**b**) NO${}_{2}$; and (**c**) NH${}_{3}$ gases as a function of concentration.

The response and recovery time were calculated from 10%–90% of the total change in output current. However, in a dynamic environment with continuous gas flow, it is not practical to wait until 90% of sensor response before taking a measurement; furthermore, in a realistic exhaust system, the gas concentration may change too rapidly for this to be a practical approach. The sensors should be able to follow small changes in concentration with a fast dynamic response. Figure 5 shows the response of the sensors for the three target gases with different exposure times to different concentrations.

**Figure 6:**
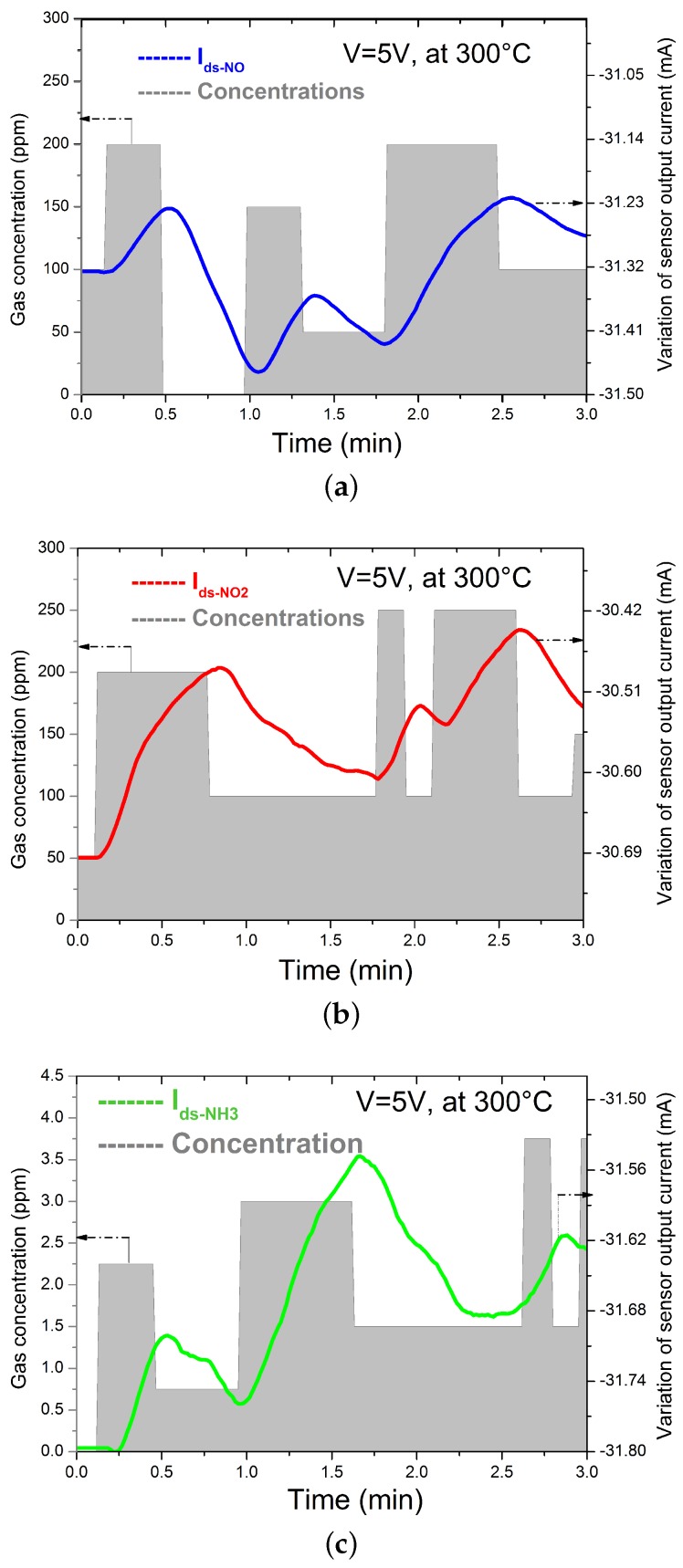
Dynamic response time of the Pt-AlGaN/GaN HEMT sensor to (**a**) NO; (**b**) NO${}_{2}$; and (**c**) NH${}_{3}$ as a function of concentration.

In this type of test, we scan the exposure time from 10 s down to 1 s for a given concentration of each gas at an operating temperature of 300 °C, and after each exposure time, we put the sensor under pure nitrogen. The sensors show the ability to detect the change of gas concentration for the shortest tested exposure time (1 s), even for small concentrations of NO (100 ppm). Contrary to NO, the sensor shows a detection limit to 100 ppm NO${}_{2}$ at 3 s, where it cannot follow the change in gas concentration for exposure time faster than 3 s. This is attributed to the explanation that the chemical reaction of NO${}_{2}$ molecules on the Pt surface is represented by two steps: dissociation and subsequent ion diffusion, which requires more time to affect the potential barrier at the Pt-AlGaN interface than the NO molecules. NH${}_{3}$ also showed limitations for fast exposure times, but only for a very small concentration (1.5 ppm). To test our sensor under comparable conditions to real exhaust requirements, experiments were performed where the gas concentration was changed at the output of a simulated exhaust line (Figure 6), where the gray rectangles represent the different concentrations for each gas and their widths represent the exposure time. The curves represent the change of the output current of the sensor $I_{ds}$. The measured current is plotted with negative values to facilitate the understanding of the measurements.

### 3.2. Performances of the Pt-AlGaN/GaN HEMT-Based Sensor at an Operation Temperature between 300 °C and 600 °C

We first observe that the sensor has the ability to follow the change of concentration even without any regeneration using nitrogen. Second, the dynamic response time, which is represented by the delay between the time when the concentration changes and the time when the sensor signal changes, is in agreement with the first measurement (can reach 1 s), which can allow for control of the antipollution system in a closed loop.

**Figure 7:**
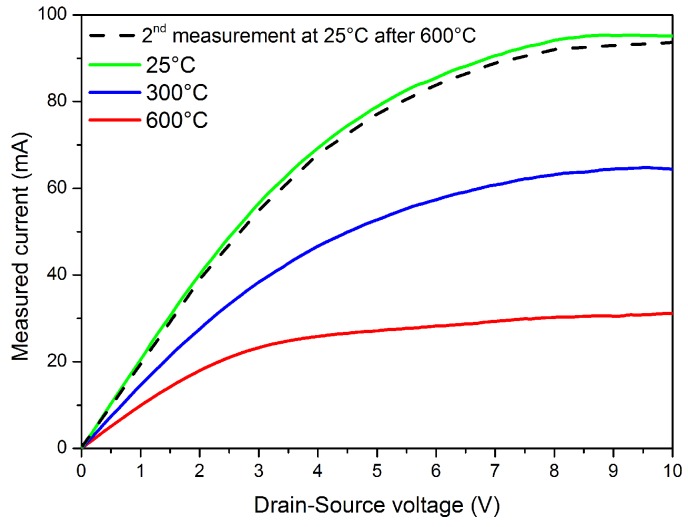
$I_{ds}$–$V_{ds}$ characteristics of Pt-AlGaN/GaN HEMTs in N${}_{2}$ for different operating temperatures.

As we mentioned in the beginning of this paper, our primary goal is to fabricate sensors that have the possibility to operate in a harsh environment, especially at high temperatures up to 600 °C and that demonstrate fast response times. In this experimental study, we operated the sensors in a wide range of temperatures, from room temperature to 600 °C. The current-voltage characteristics (*I*–*V* curves) as a function of operating temperature were measured in N${}_{2}$ carrier gas, as well as in the presence of the three target gases for different concentrations. The figure below (Figure 7) represents the effect of the operating temperature on the transistor device operation. $I_{ds}$–$V_{ds}$ characteristics were measured at various operating temperatures using drain to source voltage $V_{ds}$ equal to 5 V without any gate bias voltage ($V_{gs}$ = 0 V). In this test, we increased the temperature from room temperature (25 °C) to 600 °C with a step of 100 °C and a rate of 30 °C/min. After each step, the transistor was kept at the same operating temperature for 45 min.

The device *I*–*V* curves show that even at an operating temperature of 600 °C, we can clearly distinguish between the linear and saturation region, which is indicative of good HEMT performance. The decrease of $I_{ds}$ with increasing the operating temperature can be explained by the activation of more bulk traps and the change of the electron mobility in the 2DEG channel, which is due to the increasing of electron phonon scattering [25,26]. These good HEMT characteristics mean that there is practically no degradation in terms of structure (AlGaN/GaN heterostructure) or in terms of ohmic contacts in this range of operating temperature. After 45 min of HEMT operating at 600 °C, another *I*–*V* curve was measured at 25 °C, as shown in Figure 7 (see the dashed line), which represents nearly the same $I_{ds}$ as the first measurement and confirms the good reproducibility after the operation at high temperatures.

**Figure 8:**
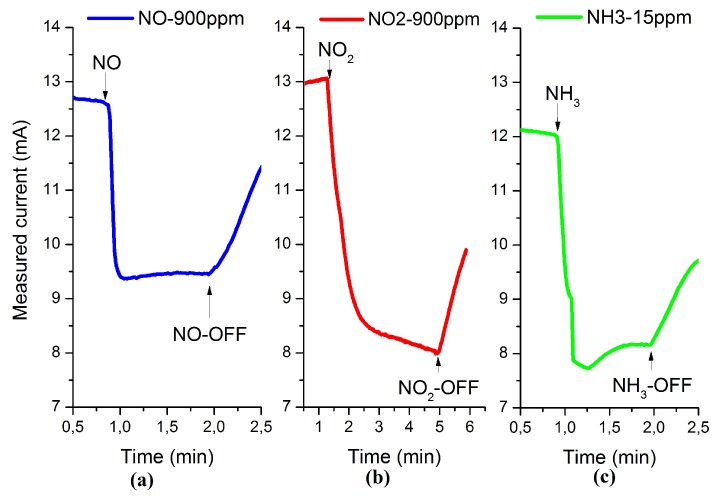
Response of the HEMT sensor to (**a**) 900 ppm NO; (**b**) 900 ppm NO${}_{2}$; and (**c**) 15 ppm NH${}_{3}$ at 600 °C.

To further investigate the temperature dependence of the sensitivity and the response time of the sensors, we repeated the same measurements using the three target gases with 900 ppm of NO, 900 ppm of NO${}_{2}$ and 15 ppm of NH${}_{3}$ over the same temperature range. Figure 8 represents the sensor response at 600 °C. The temperature scan showed that the sensitivity of the sensors to the three gases increases with increasing the operation temperature up to 600°C. The sensitivity reaches values of 24%, 38.5% and 33% for 900 ppm NO, 900 ppm NO${}_{2}$ and 15 ppm NH${}_{3}$, respectively.

**Figure 9:**
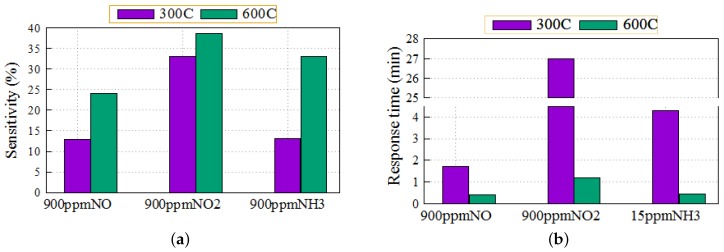
Comparison of the performances of the sensor (**a**) sensitivity; and (**b**) response time, as a function of operation temperature (300 °C and 600 °C).

This is attributed to enhanced dissociation of NO${}_{2}$ and fast diffusion of oxygen ions with increasing the operating temperature, especially after 300 °C. At the same time, increasing the operating temperature leads to an acceleration of the desorption of the gas molecules from the functional layer surface, which increases the available adsorption site at a given time. Furthermore, increasing the operation temperature leads to a decrease in both the response and recovery times for the three gases to reach 0.4 min, 1.2 min and 0.45 min for 9000 ppm NO, 9000 ppm NO${}_{2}$ and 15 ppm NH${}_{3}$, respectively, at 600 °C, as shown in Figure 9.

This decrease in response time might be due to more effective catalytic dissociation of NO${}_{2}$ and NH${}_{3}$ on the Pt layer and, therefore, the improvement of the adsorption and diffusion rate. The Table 1 summarizes the comparison of the sensor performances between the two environmental conditions.

## 4. Conclusions

We have demonstrated AlGaN/GaN HEMT-based sensors that are highly sensitive to NO, NO${}_{2}$ and NH${}_{3}$ gases. Chemical reactions between gas molecules and the functional layer (Pt gate) lead to changes in the output current of the device. The sensors tested in this study demonstrate that they can be used to detect NO and NO${}_{2}$ in a wide range of concentrations (from 100 ppm–900 ppm) with high sensitivity, along with NH${}_{3}$ detection at concentrations as low as 3 ppm. Furthermore, we demonstrate that the sensors are suitable for harsh environments, and they show high performance in terms of sensitivity and response time at operating temperatures up to 600 °C. Finally, we demonstrate that these sensors can follow small changes of the gas concentrations, even for small exposure times of 1 s. Based on the results presented in this paper, HEMT sensors are suitable for the control of diesel antipollution systems in real time.

## Figures and Tables

**Table 1:** Pt-AlGaN/GaN HEMT sensor performances as a function of the operation temperature at V_*ds*_ = 5 V.

Type of Gas	Concentration (ppm)	Temperature (C)	I_0_ (mA)	Delta I (mA)	Sensitivity (%)	Response Time (min)
NO	900	300	54.5	7	12.8	1.7
		600	12.5	3	24	0.43
NO_2_	900	300	44	14	33	27
		600	13	5	38.5	1.2
NH_3_	15	300	49.5	7	13	4.3
		600	12	4	33	0.45
